# Phantom-Mobility-Based Prosthesis Control in Transhumeral Amputees Without Surgical Reinnervation: A Preliminary Study

**DOI:** 10.3389/fbioe.2018.00164

**Published:** 2018-11-29

**Authors:** Nathanaël Jarrassé, Etienne de Montalivet, Florian Richer, Caroline Nicol, Amélie Touillet, Noël Martinet, Jean Paysant, Jozina B. de Graaf

**Affiliations:** ^1^CNRS, INSERM, Institut des Systèmes Intelligents et de Robotique, ISIR, Sorbonne Université, Paris, France; ^2^CNRS, ISM, Aix Marseille Université, Marseille, France; ^3^Centre Louis Pierquin, Institut Régional de Médecine Physique et de Réadaptation, UGECAM Nord-Est, Nancy, France

**Keywords:** prosthetics, transhumeral amputation, phantom limb, myoelectric control, pattern recognition, voluntary phantom limb mobility

## Abstract

Transhumeral amputees face substantial difficulties in efficiently controlling their prosthetic limb, leading to a high rate of rejection of these devices. Actual myoelectric control approaches make their use slow, sequential and unnatural, especially for these patients with a high level of amputation who need a prosthesis with numerous active degrees of freedom (powered elbow, wrist, and hand). While surgical muscle-reinnervation is becoming a generic solution for amputees to increase their control capabilities over a prosthesis, research is still being conducted on the possibility of using the surface myoelectric patterns specifically associated to voluntary Phantom Limb Mobilization (PLM), appearing naturally in most upper-limb amputees without requiring specific surgery. The objective of this study was to evaluate the possibility for transhumeral amputees to use a PLM-based control approach to perform more realistic functional grasping tasks. Two transhumeral amputated participants were asked to repetitively grasp one out of three different objects with an unworn eight-active-DoF prosthetic arm and release it in a dedicated drawer. The prosthesis control was based on phantom limb mobilization and myoelectric pattern recognition techniques, using only two repetitions of each PLM to train the classification architecture. The results show that the task could be successfully achieved with rather optimal strategies and joint trajectories, even if the completion time was increased in comparison with the performances obtained by a control group using a simple GUI control, and the control strategies required numerous corrections. While numerous limitations related to robustness of pattern recognition techniques and to the perturbations generated by actual wearing of the prosthesis remain to be solved, these preliminary results encourage further exploration and deeper understanding of the phenomenon of natural residual myoelectric activity related to PLM, since it could possibly be a viable option in some transhumeral amputees to extend their control abilities of functional upper limb prosthetics with multiple active joints without undergoing muscular reinnervation surgery.

## 1. Introduction

People with an upper arm amputation represent a significant part of the major upper-limb amputees in western countries (for instance 33% in France André and Paysant, 2006 and 45% in the United Kingdom National Amputee Statistical Database, 2009). These patients, most of them young active people, are usually fitted with a functional prosthesis (financed by the Social Security) composed of several active joints (hand, wrist and sometimes elbow), allowing them to regain a certain autonomy. While there have been improvements of prosthetic solutions over the last years, the situation remains particularly complex for the common case of transhumeral (i.e., above elbow) amputees. The control is difficult to learn, remains non-intuitive, and thus cognitively demanding. Moreover, these prostheses lack functionality and so do not provide the expected assistance in Activities of Daily Life (ADLs) (Biddiss and Chau, 2007). This leads to the development of compensatory strategies involving the rest of the body, causing shoulder, trunk, and contra-lateral limb disorders (Ostlie et al., 2011). The consequence is that transhumeral amputees are more likely to reject their prosthesis than transradial amputees (Wright et al., 1995; Biddiss and Chau, 2007).

One of the current major issues, common to all levels of upper-limb amputation, is the growing gap between the available hardware of arm prostheses, becoming more biomimetic with numerous active joints (e.g., polydigital hands), and their still counter-intuitive and sequential control that limit their actual use (Atkins et al., 1996). For instance, the myoelectric control, which is the most common method to command an externally-powered upper limb prosthesis, relies on the use of ElectroMyoGraphic signals (EMG) from two antagonistic muscles of the residual limb (generally the biceps and triceps). An on/off strategy is applied by thresholding the input signals [amplitude and temporal variations of surface ElectroMyoGrams (sEMG)] that the patient needs to produce with the equipped muscles. Often, each active prosthetic joint that composes the substituting limb is sequentially controlled by the same control inputs. So, despite the potential possibilities offered by the new biomimetic prostheses like whole robotic arms (Resnik et al., 2014) or polydigital hands (Belter and Dollar, 2011), their control remains complex, as it is far from intuitive, and offers few functional Degrees of Freedom (DoF) (Castellini et al., 2014).

To overcome these limitations, pattern-recognition approaches have been developed since the late 60s/70s (Finley and Wirta, 1967; Herberts et al., 1973; Lawrence et al., 1973) aiming a more precise decoding of myoelectric signals in order to improve the recognition of different muscle activation patterns and thus to control more types of movements. This requires the use of multiple recording sites, a precise extraction of signal characteristics (not only amplitude) and a multidimensional classification architecture. While well established and extensively studied in research institutions, such approaches have only very recently been applied commercially to prosthetics control (e.g., COAPT system, http://www.coaptengineering.com/).

One way of feeding pattern recognition myoelectric control is to rely on sEMG activities of the residual limb associated with phantom limb movement (PLM) execution. Indeed, voluntary PLM have recently been shown to be controlled as intact limb movements (Raffin et al., 2012a,b; Garbarini et al., 2018), with associated muscle activities that vary with the type of executed PLM (Reilly et al., 2006; Raffin et al., 2012a; Jarrasse et al., 2017b). This approach has been quite extensively studied for below-elbow amputees whose residual limb usually still contains the muscles that mobilized the fingers before the amputation, and, therefore, provide an adapted measurement site together with relatively strong myoelectric signals. While first attempts of adaptation of these approaches to above-elbow amputees date back to the 70s with pioneering work like (Wirta et al., 1978), several studies recently tried to revive such approach using updated classification techniques, specifically of phantom-limb-mobility-related EMG signals (Jarrasse et al., 2017b; Gaudet et al., 2018), even extended to individual finger movement decoding (Jarrasse et al., 2017a). Also, recent work on the treatment of phantom limb pain with help of virtual reality (e.g., Ortiz-Catalan et al., 2016) illustrates the possibility of decoding these PLM-associated EMG patterns measured on the residual limb of transhumeral amputees. Yet, after transhumeral amputation, PLM-related myoelectric activity is measured over muscle groups of the residual limb which -before amputation- were not naturally related to the missing limb (i.e., hand and wrist), and, therefore, inevitably more complex to decode. So, daily life phantom-based prosthetic control is more challenging, especially without reinforcing PLM-associated sEMG signals through muscular reinnervation surgery (Kuiken et al., 2007). One might, for example, expect an influence of the fatigue associated to PLM generation and of the remaining mismatch between the actual PLM movements and the ones generated in reaction by the prosthesis (the velocity and range of motion of these PLM being generally limited) (De Graaf et al., 2016), on the number of possible PLM that the patients can execute the one after the other.

Most studies on the development of more natural prosthetic control approaches based on PLM decoding (Powell et al., 2014; Atzori et al., 2016; Jarrasse et al., 2017b; Gaudet et al., 2018) have so far been conducted on offline pattern recognition of pre-recorded myoelectric sequences, or either using simple computer interface control of performing simple free motions using a real prosthesis. To our knowledge, no experiments involved the completion of a functional task during which the prosthesis had to be controlled using real time decoding of PLM while the participant controlled the interaction with objects. The objective of this study is thus to evaluate the possibility for transhumeral amputees to use a PLM-based control approach to actually perform a set of more realistic functional tasks with an unworn prosthetic arm with numerous active DoF (elbow, wrist rotator, polydigital hand with two different types of grasping). Such a task will challenge the precision of control and the fatigue associated to PLM execution (De Graaf et al., 2016), and will offer a first evaluation of the “interaction” between “phantom activity” and the prosthetics with tasks in the “real world”. We present in this paper the results of an experiment during which two patients had to voluntarily mobilize their phantom limb to have an arm prosthesis mimicking the phantom movement.

## 2. Materials and methods

**Figure 1:**
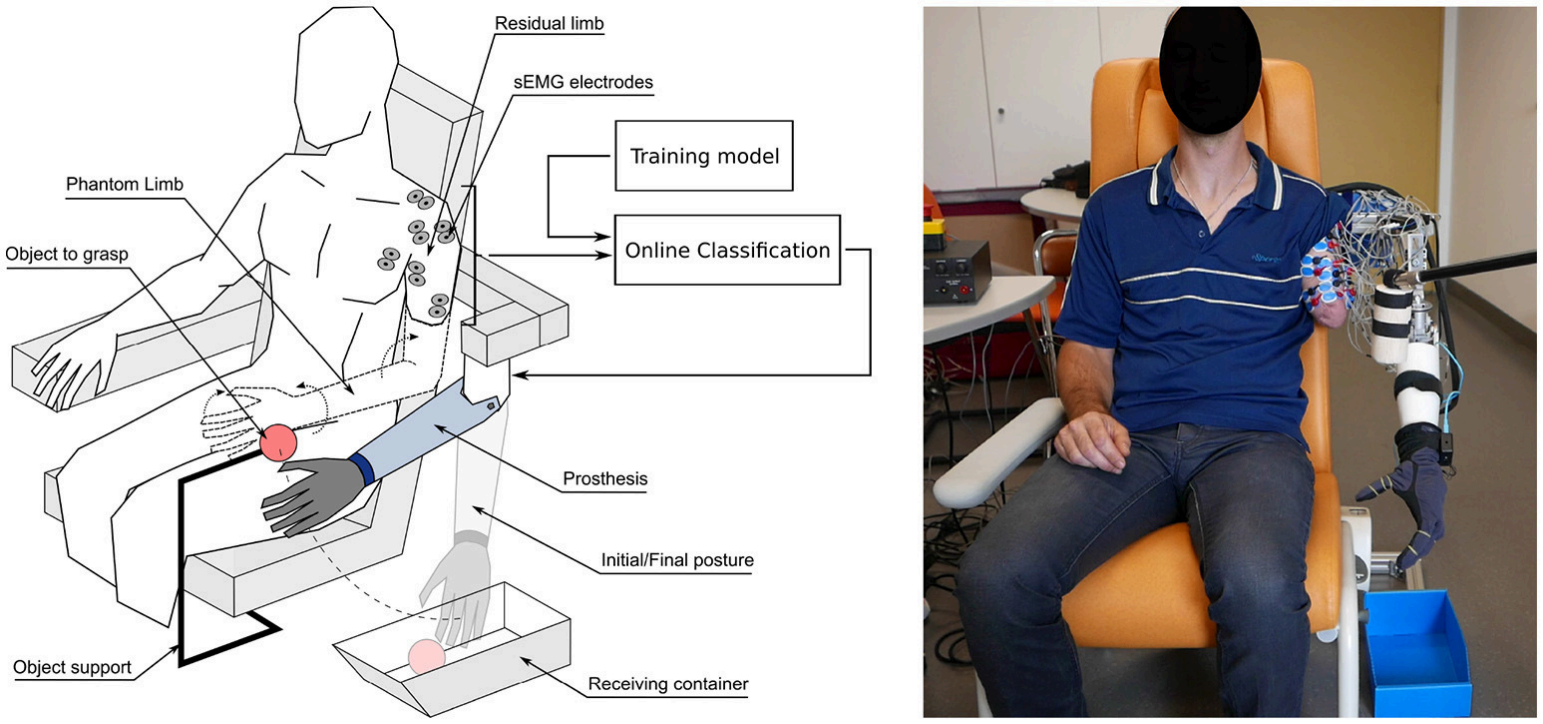
**(Left)** Global view of the experimental setup during one of the functional tasks of grasping an object (here the foam tennis ball) and releasing it in the dedicated container, with the arm prosthesis controlled through the associated mobilization of the phantom limb. **(Right)** Photo of the setup being used with P2.

The prosthesis (Figure 1) was composed of an active elbow, a wrist rotator and polydigital hand, attached to a fixed support placed close to the residual limb. A direct control mapping was used to associate a phantom movement of one joint to a movement of the same prosthetic joint. Eight different movements of the elbow, wrist (rotation), hand and pinch were used within this experiment. We first evaluated the pattern recognition architecture performance in amputees, and then analyzed the grasping task performance through different metrics (timing, optimality of sequences, kinematics) in comparison with performance obtained by three non-amputated (control) participants performing the same task with a simple computer interface.

### 2.1. Participants

Since this is a preliminary study, a limited number of participants were tested. Two participants (in the 35–55 age range) with a unilateral transhumeral amputation of traumatic origin were selected to participate to the study. Three healthy participants (in the 25–27 age range) were recruited as a control group to perform the grasp and release task through a simplified computer GUI control. This study was carried out in accordance with the recommendations of the Université Paris Descartes ethic committee CERES (N°IRB 20151900001072), which had approved the protocol. All participants provided written informed consent to participate in the study, and both patients gave written permission for publication of photographs for scientific and educational purposes. The protocol was performed in accordance with the Declaration of Helsinki.

The selection of the two amputated participants was based on the level of amputation (only transhumeral amputees), their control ability of a mobile phantom limb (i.e., the possibility to perform several different types of phantom movements), the absence of phantom and residual limb pain, and the availability of the participants during the recording period. The first participant (P1), because of an injured plexus brachial leading to limited muscle contraction amplitudes, has never been fitted with a myoelectric prosthesis. On the contrary, the second participant (P2) is daily using a myoelectric prosthesis composed of a motorized Utah^©^ elbow, an active wrist rotator and a polydigital hand (iLimb Ultra, Touch Bionics^©^), controlled by two electrodes placed over his biceps and triceps muscle (the control-switching between joints being achieved through a co-contraction of both muscles). Table 1 resumes demographic data.

**Table 1:** Demographic data concerning the 2 amputated participants.

**Participant**	**Elaps. time**	**Amput. side**	**Dom. ?**	**Amput. type**	**Amput. cause**	**Pain treat**.	**Prosthesis**
P1	34 years	Left	No	1/3 arm Left	traumatic	No	None or aesthetic
P2	4 years	Left	No	2/3 arm Left	traumatic	No	Myoelectric

*M, male; Dom. ?, Dominant limb prior to amputation; Elap. time, Elapsed time since amputation. “Pain Treat.”, pain treatment. “Prosthesis” indicates the type of prosthesis the patients usually wears*.

The amputated participants were followed-up at the Louis Pierquin Centre of the Regional Institute of Rehabilitation, Nancy, France. Their voluntary mobilization of phantom limb had been explored through a questionnaire and a preliminary evaluation in order to make clear distinctions between residual limb sensations, phantom pain, phantom sensations, and most importantly, between mobility of the residual limb and that of the phantom limb (Touillet et al., 2018). Both participants reported a good feeling and control of their phantom hand, including separate whole hand and pinch (thumb and index) opening and closing, wrist rotation and flexion/extension of the phantom elbow.

### 2.2. Experimental setup

#### 2.2.1. Upper limb prosthetic platform

**Figure 2:**
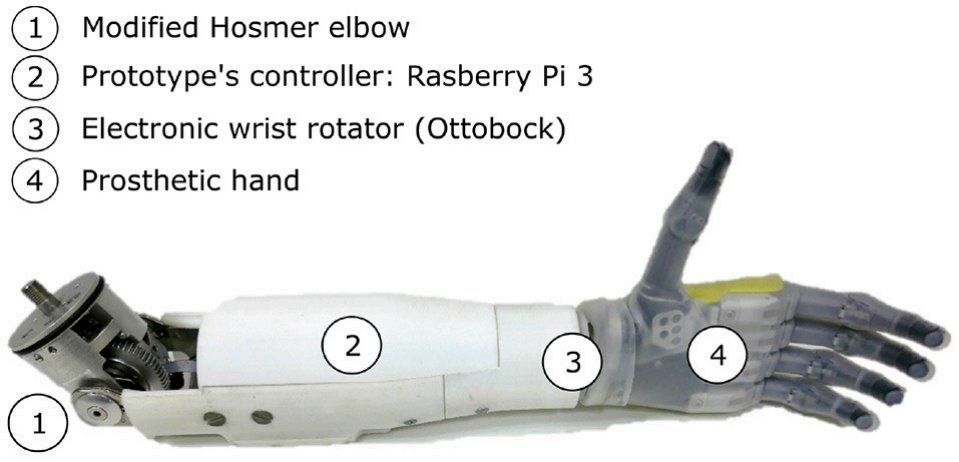
The arm prosthesis prototype includes a motorized elbow (1), an embedded controller based on a Raspberry Pi 3 (2), an electronic wrist rotator (3), and a Touch Bionics Robolimb (4).

The upper limb prosthetic used for this experiment (see Figure 2) was designed using modified commercially available prosthetic parts: a Fillauer^©^ Hosmer E-TWO electric elbow, a conventional electric wrist rotator from Ottobock^©^ (model 10S17), and a Robolimb polydigital hand from Touch Bionics^©^ possessing 6 active joints (one per finger flexion plus the thumb rotation). This prototype was attached directly to a dedicated support frame made of aluminum profiles, which was adjusted to place the prosthetic elbow close to the phantom elbow joint position. The overall prosthetic system was controlled by an embedded Raspberry Pi 3 which drives the elbow and wrist joints through a dedicated position/velocity motor controller (Roboclaw Motor Controller from Ion Motion Control^©^), and the polydigital hand through a generic serial connection. A dedicated C++ program was developed (running at a frequency of 100 Hz on the Jessie OS^©^ from Debian Linux^©^) to receive movement instructions (received from a wifi socket connection), to control the active joints accordingly (with respect to a set of predefined parameters like joint velocities), to monitor the kinematic activity and to store recorded data in files.

To monitor the kinematic activity of the upper-limb prosthetic, a motion capture dataglove (VMG 30 from Virtual Realities LLC^©^ relying on piezoelectric technology) was placed over the prosthetic hand. Thanks to a dedicated calibration phase (performed once for each patient), the prosthetic fingers activity were recorded at 25 Hz. This glove was also fitted with two Inertial Measurement Units (IMU, 9 degrees-of-freedom) allowing us to track both the elbow flexion and wrist rotation kinematics at a similar frequency.

Based on previous individual recordings of intact arm kinematics when mimicking their PLM (De Graaf et al., 2016), the joint velocities of the prosthesis were pre-set as follows: 15°/s for the elbow flexion/extension velocity (slow compared to the natural adopted 105°/s measured in healthy subjects Farthing and Chilibeck, 2003), 40°/s for the wrist rotation and 2.6 s for complete opening or closing the whole hand.

#### 2.2.2. Surface EMG recordings from the residual limb muscles

**Figure 3:**
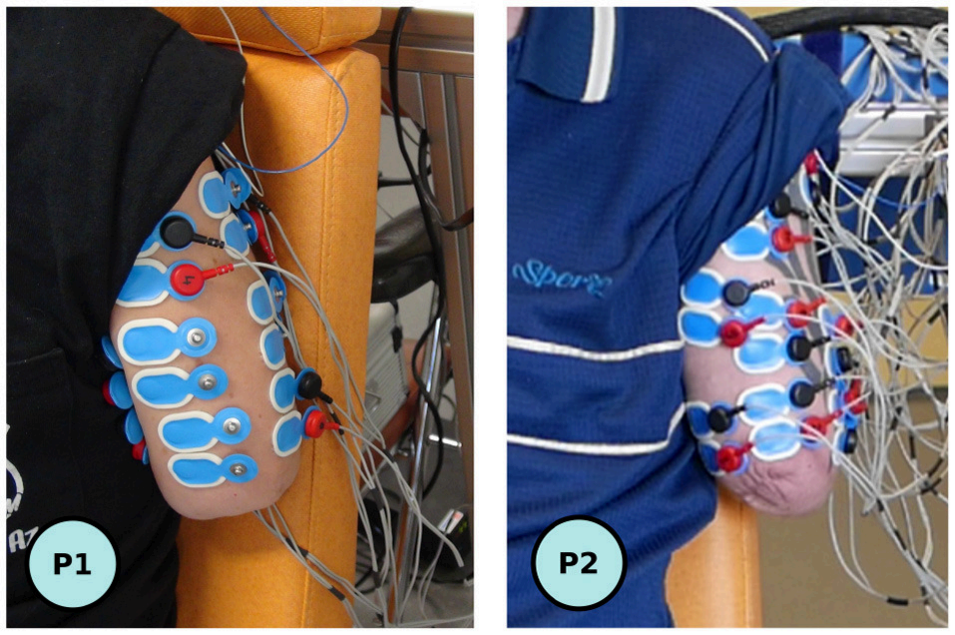
View of the residual limbs with the connected six optimal (P1, **left**), respectively initial twelve (P2, **right**) pair of electrodes.

A dedicated electrophysiological signal-recording system (Eegosports from ANT-Neuro^©^, The Netherland) with bipolar shielded channels at 24-bit resolution was used to record sEMG muscle activities from the participant's residual limb at a 1 kHz frequency. Because of the variability in residual limb length and muscle anatomy due to the level of amputation, the scheme of electrodes placement had to be adapted for each participant (see Figure 3). Twelve pairs of sEMG electrodes (Ambu^©^ BlueSensor Ag/AgCl snap bipolar electrodes with a 1.25-cm-diameter circular contact and a 2 cm inter-electrode distance (center point to center point) were initially placed on each participant to measure activity on various parts of the residual biceps, triceps, deltoid and sometimes trapezoidal and pectoralis major muscles. No specific skin preparation was used before placing the active electrodes on the residual limb.

The recorded sEMG signals were then filtered with a [10 Hz ; 400 Hz] third-order bandpass Butterworth filter and a notch filter to remove the power line 50 Hz noise (Q factor of 35). A filter approach exploiting the properties of the Principal Component Analysis (derived from Kvas and Velik, 2008) was then applied to the first sEMG recordings for the training of the pattern recognition algorithm. This analysis resulted in the initial selection of an optimal set of 6 pairs of electrodes (maximizing the classification results) among the 12 channels initially placed. Once this optimization was performed, the set was used for the whole experimental session.

#### 2.2.3. Classification architecture

**Figure 4:**
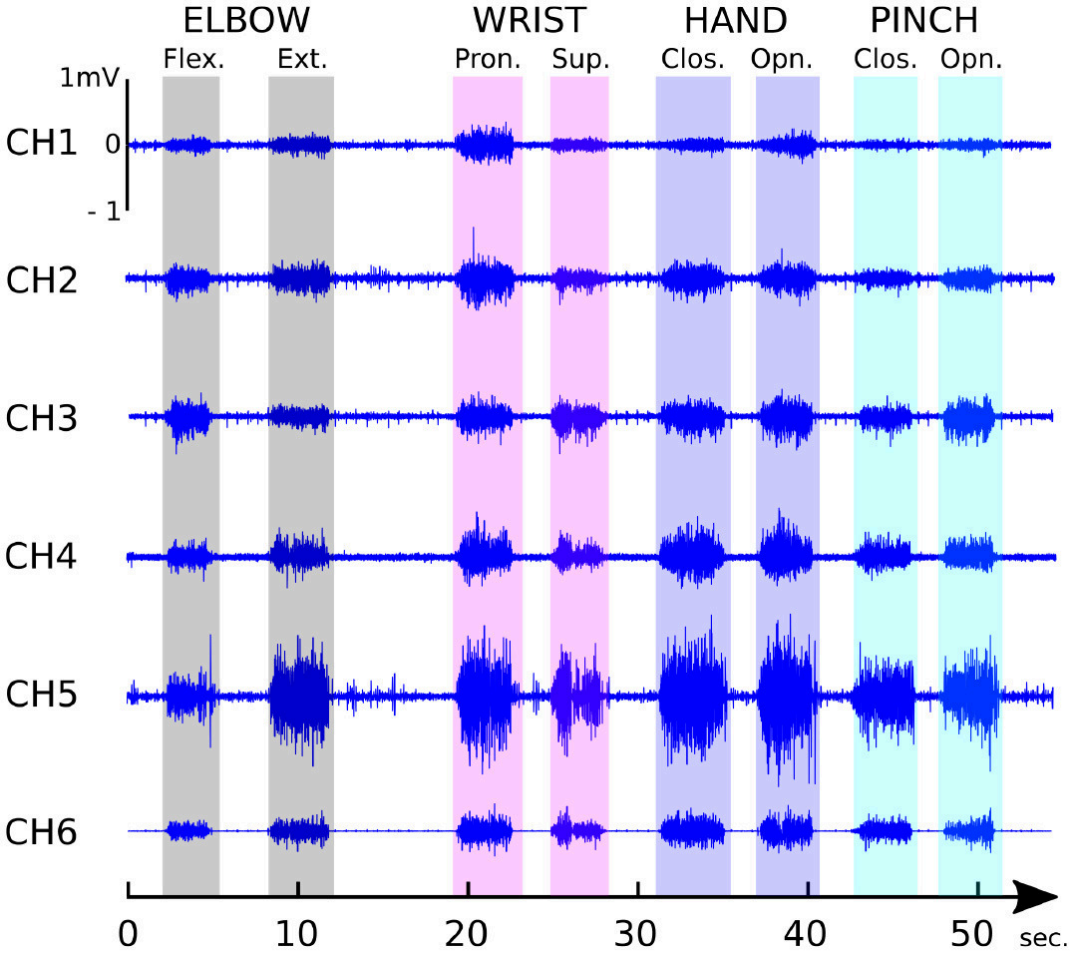
P1 typical sEMG patterns associated to the voluntary mobilization of the phantom limb recorded by the six selected electrodes when performing successively 8 different phantom limb movements.

As shown on Figure 4, the classification of given phantom limb movements cannot only rely on amplitude analyses of sEMG bursts, a method often used for the study of voluntary limb movements with well defined sEMG electrode placement according to SENIAM recommendations (Hermens et al., 2000). Additional features of sEMG signal characterizing its frequency and complexity aspects are needed for optimal classification. We used the BCI2000 software suite to develop a global control architecture on a desktop computer running Windows 7 (Intel Core i5-4690K (3.5 GHz) with 16 Go DDR3). BCI2000 is a general-purpose software suite designed for brain-computer interface (BCI) and was used here to run in parallel three principal modules: one acquisition driver to acquire the sEMG data (at a 1 kHz frequency), one Matlab classification algorithm script (executed every 128 ms), and one graphical user interface (C++ with Qt) which was also broadcasting in real time the classification output to the network, in order to transmit the kinematic instructions to the prosthesis. A generic Linear Discriminant Analysis (LDA) classifier (Englehart et al., 1999) running on Matlab^©^ (relying on the “fit discriminant analysis classifier” function of the Statistics Toolbox) was used to classify the myoelectric activities. The features were computed from the sEMG using a 512-ms-sliding analysis window with a 128-ms-overlap between successive windows. Among the wide variety of features that have been investigated in the literature (Phinyomark et al., 2013), we selected those known to be the most efficient and robust for the classification of sEMG with LDA: the root mean square (RMS) value (Oskoei and Hu, 2008), the first 4 autoregressive coefficients (AR) (Zardoshti-Kermani et al., 1995; Tkach et al., 2010), the zero crossing and the sample entropy (Richman and Moorman, 2000) of the sEMG were extracted from each channel and used to create the feature vector. No dimensionality reduction nor post-processing methods was used. Only the confidence value of the classifier was used to filter the algorithm output: if classification confidence was below 95%, no movement instruction was sent to the prosthesis.

### 2.3. Protocol

#### 2.3.1. Amputees' protocol

The participant was comfortably seated in a dedicated chair, fitted with an armrest on the non-amputated side and a head rest. The arm prosthesis, attached to its rigid support, was placed approximatively at the location of the phantom arm of the subject (see Figure 1). Once the electrodes were placed on the subject's residual limb, the session started with a training phase, was then followed by a phase of preliminary assessment of the classification and PLM-based prosthesis control, and ended with the object grasping task. During all three phases, the sEMG signals were recorded and the whole recording session was videotaped and lasted for about 120 min for each participant. The three protocol phases will now be detailed.

##### 2.3.1.1. Training of the classifier

The participant was asked to successively perform once 8 selected phantom movements (8 movements: Elbow Flexion (EF) and Extension (EE), Wrist Pronation (WP) and Supination (WS), hand, resp. Pinch Closing (HC, resp. PC) and Opening (HO, resp. PC) with a few seconds of rest after each movement. This sequence was repeated once to get 2 demonstrations per movement. The experimenter was in charge of verbally asking the subject to execute a given movement and thus determined the rythm of the performance. No instruction was given about the amplitude and the velocity of the gesture, only the need of repeatability was mentioned. It is important to highlight that this brief initial individual training of the classifier was then used for the whole experiments.

##### 2.3.1.2. Preliminary assessment of the classification and PLM-based prosthesis control

After a rapid data treatment, the training phase of the classifier was followed by a preliminary assessment of the classifier performance. During this phase, while the online classification algorithm was running, the participant was asked to induce given movements of the prosthesis through the phantom limb mobilization. The participant performed a randomized set of PLM (12 repetitions of the 8 selected gestures) during which the movements of the prosthesis reflected the PLM that were detected by the classifier. Once the movement instruction provided by the experimenter, the participant had 5 s to perform the task. Within this time period, a failed trial could be followed by a second one. Once a trial was considered as successful, or when it lasted more than 5 s, the experimenter asked the participant to relax before giving the next PLM to perform. In order to avoid incompatible successions of actions, a pseudo-randomized list of 12^*^8 for P1 (reduced to 6^*^8 for P2, see results) PLM was performed with intermediate resting periods of a few minutes every 20 movements.

##### 2.3.1.3. Grasping task

**Figure 5:**
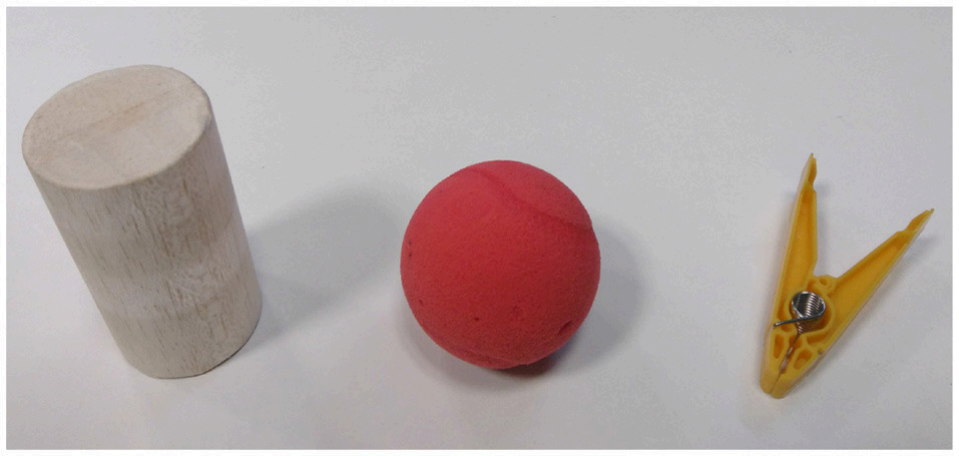
The objects used for the grasping task. From the left to the right, a cylinder made from Balsa wood from the kit of objects from the SHAP (Light et al., 2002) (diameter 60 mm, weight 30 g), a compliant foam tennis ball (diameter 70 mm, weight 12 g), and a clothespin from the Rolyan Graded Pinch Exerciser kit (model Yellow, 1 lb Pinch Exerciser, weight 20 g).

After 10–15 min of passive recovery, the protocol ended by a grasping task performance with the arm prosthesis that included a randomized set of 3 grasping repetitions of 3 different objects (shown in Figure 5 through the same control mode.

The chosen object was placed such that it could be caught by the prosthetic hand (i.e., position within the circle centered on the elbow prosthesis and which radius was equal to the hand/elbow length). The object had to be reached, grasped, detached from its support (velcro fixation requiring approximatively a 3 N force to be detached), brought back to the extended-arm posture and released in a dedicated bucket. Before the start of each trial, the prosthesis was automatically brought back to a standardized initial posture, i.e., elbow fully extended, wrist in pronosupination with the hand palm aligned with the saggital plane, and fully open hand. The final/release positions was similar, i.e., elbow fully extended and hand opened but without particular constraint over the wrist orientation. The participants had the opportunity to perform two trials with each object to train before the real grasping task phase started. The object presentation was not randomized and always started with the simplest object to grasp (cylinder) and finish with the toughest (clothespin).

#### 2.3.2. GUI control by healthy participants

In order to appreciate the performance obtained by the patients with such a control and setup, which remains very different from a natural limb control (especially because it does not allow simultaneous movements), the three healhty participants were asked to perform the exact same experiment but with the prosthesis being controlled through the use of a graphical interface on a computer screen and a mouse (each of the eight movements of the prosthesis being activated by a specific button on the graphical interface). So, no classification of EMG was necessary to control the prothesis.

### 2.4. Metrics

The online performance during the preliminary assessment of the classification and PLM-based control was analyzed using a Matlab^©^ script to automatically determine within the 5 s interval allocated to each task (i.e., performing one specific gesture with the prosthesis), the exact starting time of the participant action (when the classifier detected a change from the inactivity with a confidence over 95%) and its end (last time instant of the classifier output detection of an activity until the end of the 5 s time period). The confusion percentages were then calculated over these previously selected times of action, based on the ratio between the time of action and the time during which the correct movement was performed.

For the grasping tasks, the prosthetic arm joints were extracted from the worn motion capture glove: the embedded wrist IMU was used to compute both elbow flexion and wrist prono-supination angles. Full elbow extension was defined as a null flexion angle, while the palm parallel to the saggital plane (with the thumb pointing forward) defined the 0°posture for the wrist prono-supination. The hand closing level was averaged by the closing level of the three last fingers (from medium to little). The pinch closing level was defined as the closing level between the thumb and the index (which came into contact at around 30% of the pinch closing level because of the associated thumb rotation).

Task duration was defined as the time elapsed since the first activation detection by the control architecture (different from the “resting state”) until the object impact after falling into the receiving container.

## 3. Results

### 3.1. Preliminary evaluation of classification performance and PLM-based control

**Figure 6:**
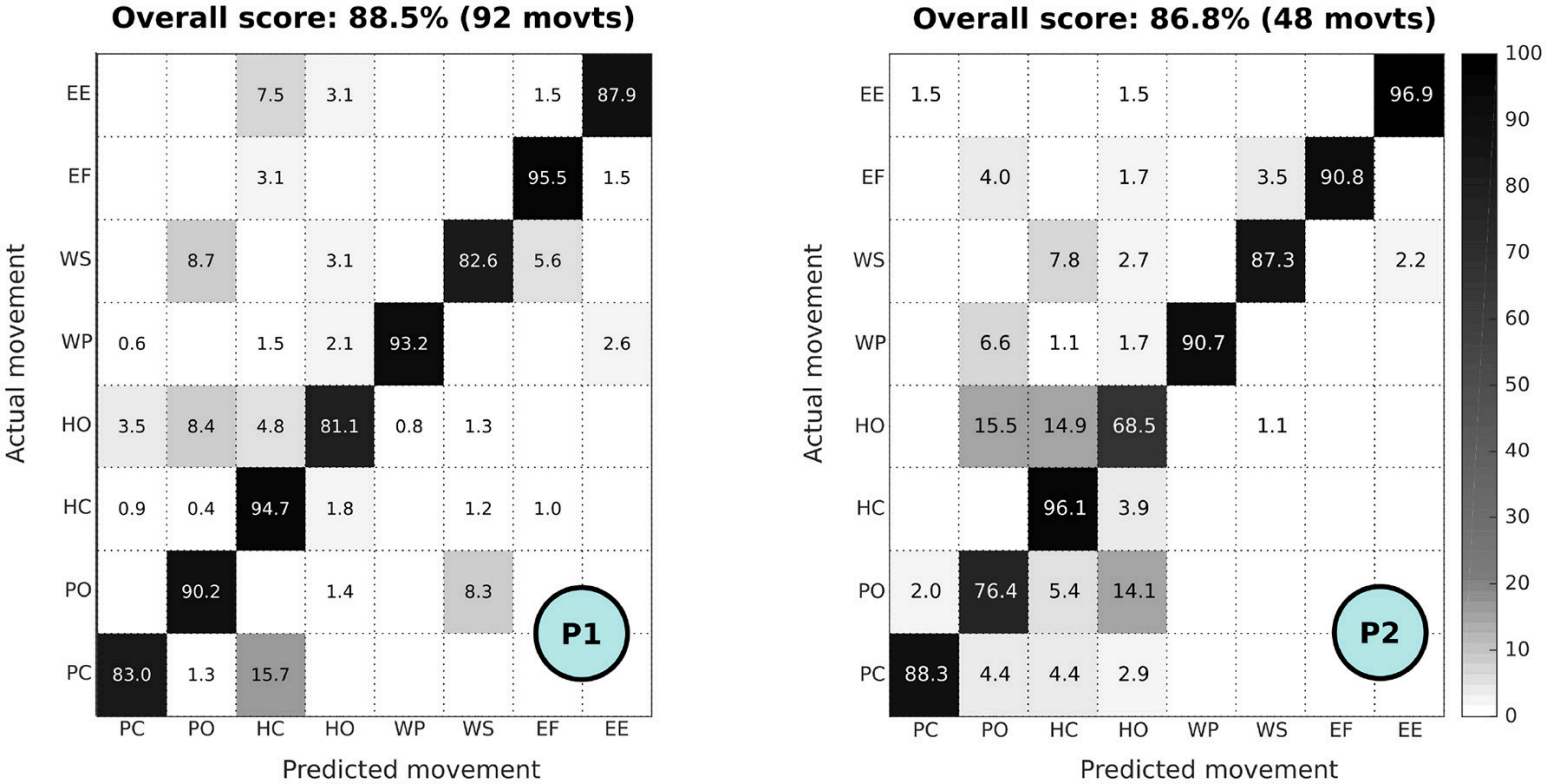
Confusion matrix of online control of the prosthesis for P1 (12 repetitions of the 8 movements, i.e., 92 movements, performed) and P2 (6 repetitions of the 8 movements, i.e., 48 movements, performed). Confusion matrix color scale is normalized across methods and increases from white to black as a function of increasing classification rate.

The confusion matrices for the participants to the preliminary evaluation of online control of the prosthesis are shown in Figure 6, indicating an averaged successful recognition score of 88.5% for P1 and 86.9% for P2. The amputated participants performed, respectively, 12 and 6 repetitions (since P2 was subject to muscle fatigue, the number of repetitions was dropped by half) of the 8 different movements of the prosthesis. For P1, only 92 movements were considered (4 movements among the 96 performed were removed because of some misunderstanding of the participant, invalidating the action). Principal confusions can be observed, respectively, between hand and pinch closing and opening: for P1, 15.7% of the pinch closing actions are considered as hand closing actions, and 8.4% of the hand openings are confused with pinch openings; for P2 rather large confusions exists between hand opening closing (14.9%), and also between hand and pinch opening (15.5 and 14.1%). Interestingly, these pinch/hand confusions were consistent with the sensations reported by the participants who indicated that they had difficulties in preventing the enrollment of the three other phantom fingers when performing pinch actions.

### 3.2. Grasp and release task performances

#### 3.2.1. Performance overview

**Figure 7:**
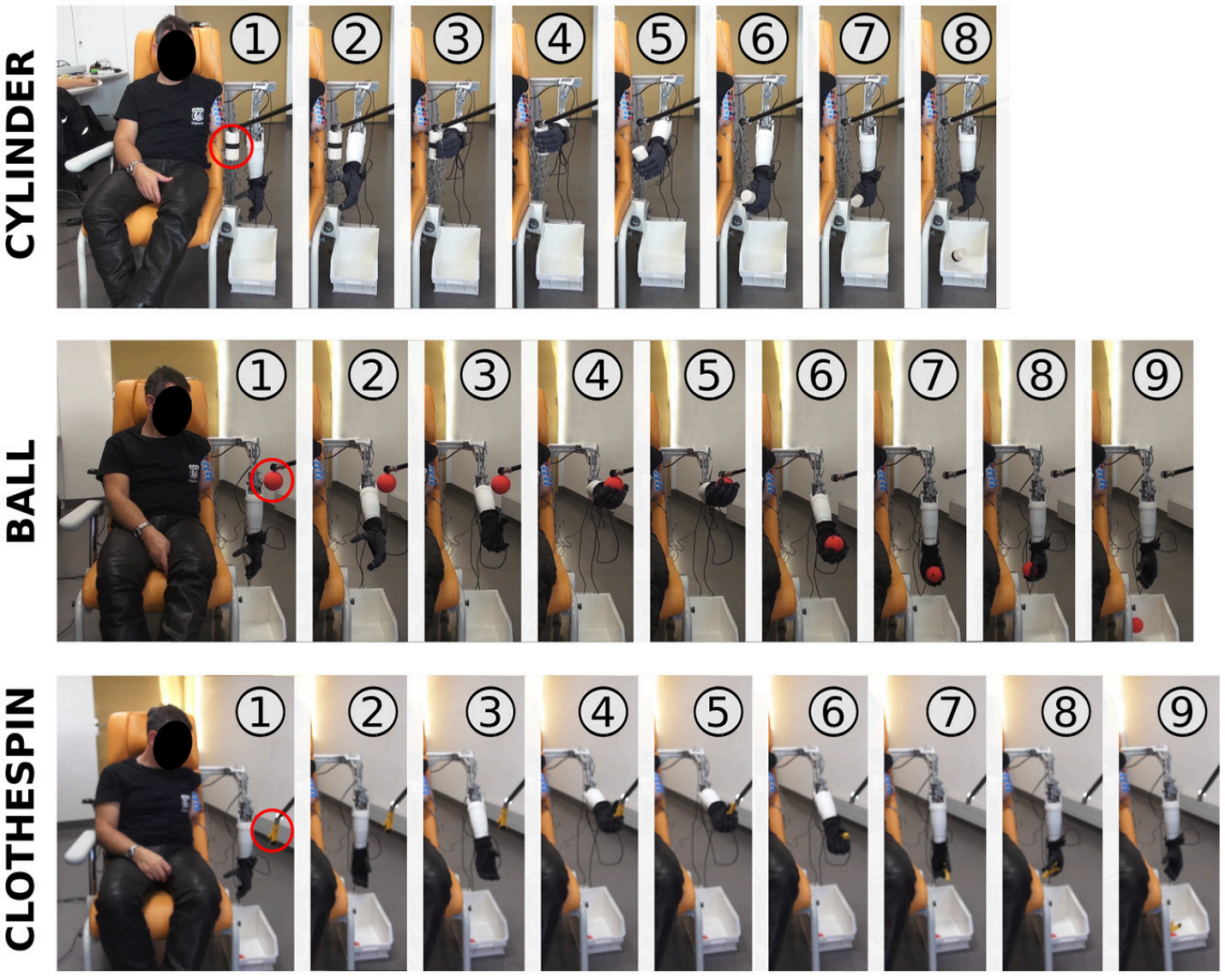
Visualization of representative grasping sequences of P1.

**Figure 8:**
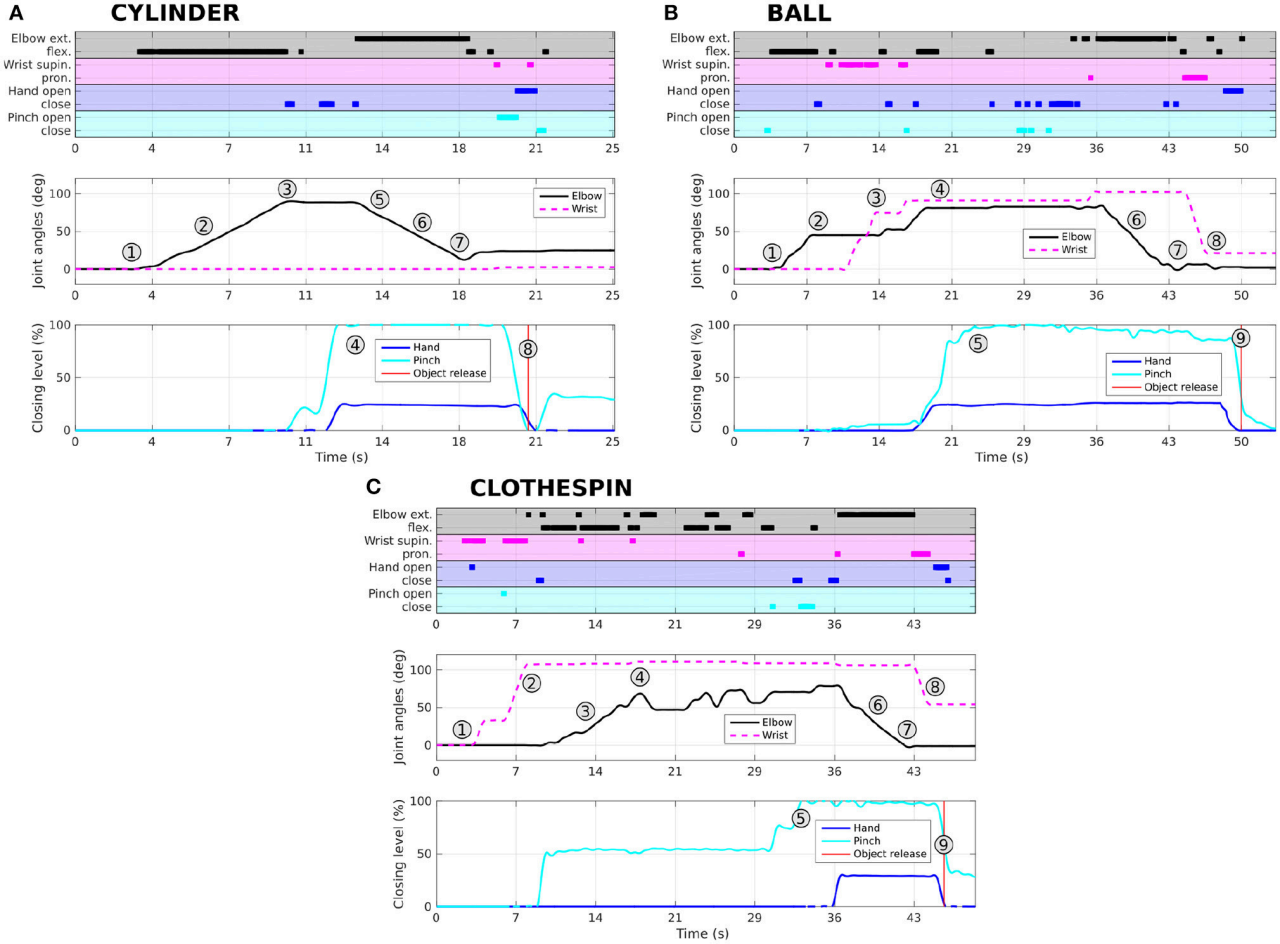
Representative profiles (associated to the 1–9 action indexes shown on Figure 7) of participant P1 for grasping and releasing the three objects (**A**: cylinder, **B**: ball, **C**: clothespin). Recognized phantom movements (output of the classification algorithm) are shown for each task, along with the associated measured kinematic variations of the 4 joints: elbow and wrist angles, and percentages of closing of hand and pinch. The action indexes (from 1 to 9) are related to the similar indices shown on Figure 7.

Representative scenarios of participant P1 (one of the three repetitions) of the three grasping tasks are shown in Figure 7. The profiles of joint kinematics and the associated classification output are shown in Figure 8. For the cylinder and ball, the participant P1 was able to perform the task following generic grasping strategies without performing numerous unnecessary and parasitic motor actions with the prosthesis. For the clothespin, he had more trouble stabilizing the elbow at the correct height (with numerous control jumps in the classifier output) to be able to grasp it (Figure 8C). As shown on the joint position profiles of Figure 8, the kinematics of the prosthetic joints is directly impacted by the classifier output changes, so even a limited (in time) misclassification leads to rather important oscillations of the prosthesis (especially at the elbow joint, due to the lever arm effect on the hand position) which perturbed the task and delayed its completion.

#### 3.2.2. Task duration

**Figure 9:**
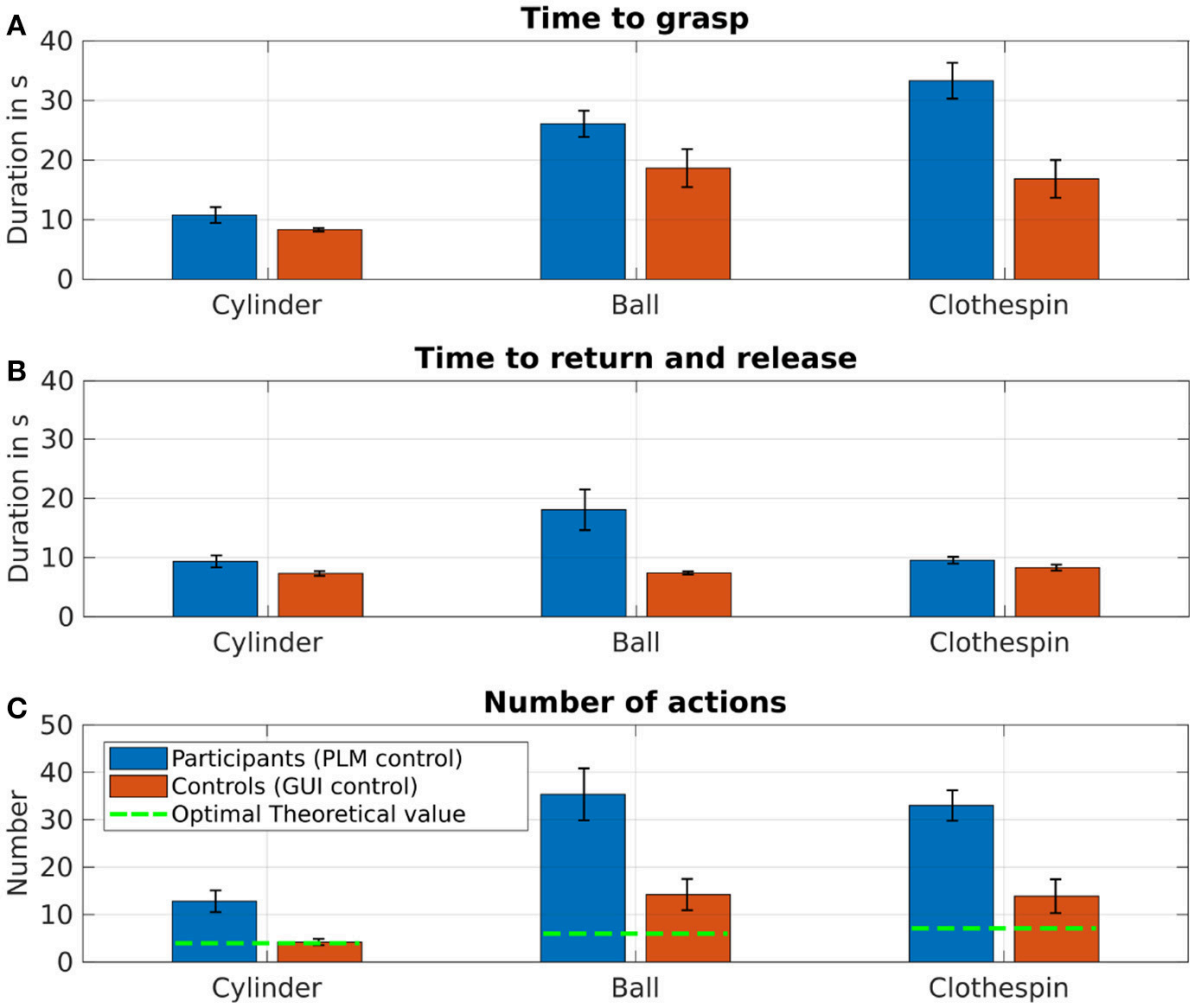
**(A)** Averaged time (± standard error) to grasp the three different objects for the amputated participants controlling the prosthesis with their phantom limb (blue), as well as for the healthy participants by sequential control through a dedicated GUI (red). **(B)** Averaged time (± SE) to return and release the three different objects for the two groups. **(C)** Averaged number of actions for completing the 3 “grasp and release” tasks.

The duration of the first grasping phase and the total task duration (time to grasp, bring back, and release the object) are shown in Figures 9A,B, for each of the three objects averaged over the two amputated participants using their prosthesis, and over the 3 control participants performing the task by sequential control through the GUI. The first phase durations for the patients are globally longer than those obtained for the healthy participants. For the cylinder the difference is small (average of 11.3 s for the amputees vs. 8.3 s for the controls), but for the ball (26 s vs. 18.7 s) it is longer, and even more for the clothespin (32.5 s vs. 16.8 s), the latter requiring a precise positioning of the fingers through precise manipulation of elbow and wrist. In comparison, the return and release times are shorter (except for the cylinder) and are showing little discrepancies between the two groups (see Figure 9B). Interestingly, the return and release of the ball required an extended time for amputated participants (18 s instead of 7.4 s). Part of the reason is that these participants at the end of the return movement added a wrist rotation before opening the hand (as shown in Figure 7 with the action 8) in order to prevent a possible bouncing of the ball outside of the container whereas the controls did not bother about that.

**Figure 10:**
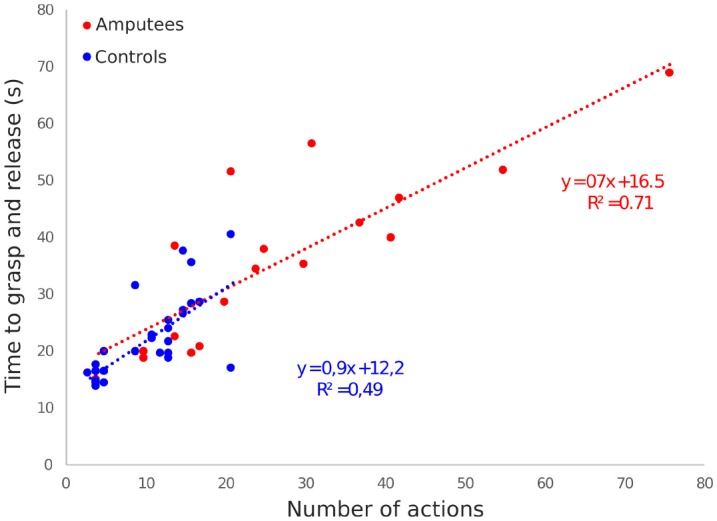
Total grasp-and-release time as a function of the number of actions needed to complete the task for all trials and objects and for each patient (in red) and healthy control (in blue). Each symbol represents one trial. The best fitting linear relation and their equations use the same color code.

Although the total grasp-and-release time is shorter for the controls than for the patients, the relation between the total grasp-and-release time and the needed number of actions to complete the task is rather similar. Indeed, Figure 10 shows the equations of the best fitting linear relation is close for the two groups.

#### 3.2.3. Averaged kinematic profiles

**Figure 11:**
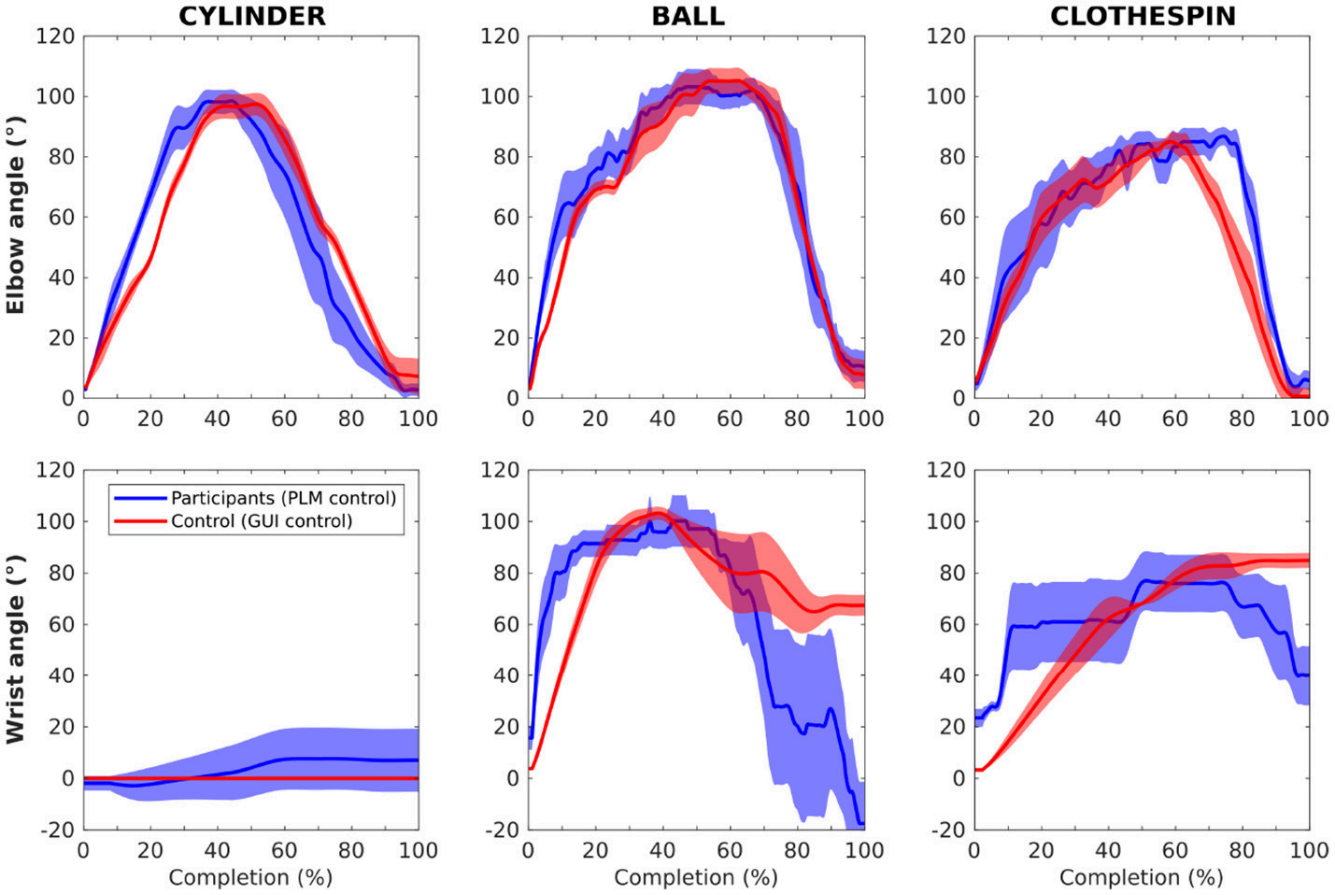
Plots of the averaged joint kinematic profiles of both elbow and wrist joints, normalized in time and averaged between repetitions and participants for the amputated (blue) and control (red) participants, and the three objects. Standard error is represented by the transparent envelopes around the curves.

Figure 11 presents the averaged joint kinematic profiles of both elbow and wrist joints, normalized in time and averaged between repetitions and participants for the two groups and the three objects. It can be seen that, independently of the durations, a similar elbow path strategy is used by the two groups, with a straight flexion extension for the cylinder, a visible intermediate step for the wrist adjustment for the ball, and a limited flexion for the clothespin which was grasped by the prosthetic finger tips and not its palm. The wrist path differed between the two groups (along with the variability within groups, more pronounced in amputees), with unnecessary wrist rotation appearing in the grasping of the cylinder by the amputated participants (possibly due to pattern recognition errors), a more segmented path for the clothespin, and, for the two last objects, no return to the initial wrist orientation for control participants. As expected, the trajectories of the control group are smoother than those of the amputated participants, essentially because of the instability of PLM control.

#### 3.2.4. Optimality of control sequences

The optimal sequences of motor actions to perform the grasping-releasing tasks of the three objects are shown in Table 2. In order to evaluate the quality of the prosthesis control in performing these three tasks, we calculated the averaged number of sequence steps used by the amputees (phantom limb control through classification) and the healthy participants (“button control”). The prosthesis control is considered as optimal when the sequence number corresponds to the theoretical minimal number of sequences listed in Table 2. Any increased number therefore can be due to a control error (possibly because of an erroneous classification), to a discontinuous movement (for example an elbow flexion discomposed in several flexion submovements), or to an alternative sub-optimal motor strategy. This index actually gives an overview of the global performance of both groups of participants and the control architecture (LDA classifier) since it is more likely to quickly increase with a classification architecture rather than with a direct control.

**Table 2:** Optimal sequences of motor action for completing each grasp and release task.

**Objects**	**Balsa cylinder**	**Foam ball**	**clothespin**
Optimal sequences	Flex the elbow and stop it at the final height	Supinate the wrist to orient the palm facing the top	Supinate the wrist to orient the palm facing the top
	Close the hand over the object	Flex the elbow and stop it at the final height	Flex the elbow and stop it at the final height
	Fully extend the elbow	Close the hand over the object	Correct the wrist orientation to face the clothespin with pinch
	Open hand to release object	Fully extend the elbow	Close the pinch over the object
		Pronate the wrist backto initial orientation	Fully extend the elbow
		Open hand to release object	Pronate the wrist backto initial orientation
			Open hand to release object
Optimal number of sequences	4	6	7

The amputees control of the prosthesis was suboptimal compared to the control group performance (11 actions vs. 4 for the cylinder, 35 vs. 14 for the ball and 33 vs. 14 for the clothespin) (Figure 9C). For the two latter objects, even the healthy subjects were not able to fully optimize their sequence of gestures (mean of 14 sequences instead of the optimal values of 6 for the ball and 7 for the clothespin, respectively).

## 4. Discussion

Two transhumeral amputated participants have been asked to repetitively grasp three different objects with an unworn active eight-DoF prosthetic arm and release them in a dedicated drawer, thanks to a prosthesis control based on phantom limb mobilization and myoelectric pattern recognition techniques, with a classifier trained using only two repetitions of each PLM. The participants successfully achieved the tasks, even if the completion times were increased (in comparison with the performances obtained by a control group using a simple GUI control) and the control strategies required numerous corrections.

The recognition rate averaged over all movements revealed to be rather high (on the average 86.4% for the two amputated participants) indicating that they were able to control 8 different prosthetic movements with a limited set of 6 electrodes placed over their residual upper-arm limb. The main confusions appeared between the hand and pinch actions. However, this confusion did not directly alter the grasp and release performances since even the precise clothespin grasping could be performed with the whole hand instead of a pinch.

Although successful, this online recognition and control rate remains slightly lower than the latest classification rate of over 90% reported in the literature (Al-Timemy et al., 2013; Farina et al., 2014). Yet, first, these latter results were obtained on transradial amputees mobilizing their residual hand and wrist muscles. It is noteworthy to recall that the present study was performed with transhumeral amputees, for whom the phantom limb phenomenon is probably related to cortical and/or neuromuscular reorganization after the amputation (Wu and Kaas, 2000; Qi et al., 2004; Gagné et al., 2011), making it more unstable compared to transradial phantom limbs. Indeed, after transradial amputation, depending on the level of amputation (the length of the stump directly conditioning the degree of presence and usability of residual extrinsic hand muscles), the muscles involved in finger, hand and wrist actions can still be present. In that case, the neuromuscular reorganization is probably less important. Second, none of these two participants mobilize their phantom limb in their daily living activities, so the used PLM were fully unusual and untrained. One might expect that training of PLM-execution probably stabilizes the associated sEMG patterns, reduces cognitive fatigue and finally improves the robustness of this control approach. Such improvements have indeed been recently reported for the control of PLM of forearm amputees (Powell et al., 2014).

This recognition rate of the PLM still allowed the two amputated participants to successfully perform the functional grasp-and-release tasks. And this remains a rather encouraging performance especially when considering the limited training of the participants compared to the several days (or weeks) sessions usually required in upper-limb amputees to master a simple (but constraining in terms of muscular contraction amplitudes) myoelectric control of active wrist and hand prostheses. Their kinematic strategies (as shown in Figure 11 and action-to-time ratio (as shown in Figure 10 were rather similar but the durations for the two complex grasps (ball and clothespin) were extended in comparison with those obtained by the control participants in their simplified -but still sequential- control task. When looking into detail the movement sequences of the prosthesis, it appeared that the major task difficulty lies in the grasping part of the task, which is not surprising since the grasping is only possible with a precise positioning of the hand. The return and release phase increased in amputees because of an additional wrist reorientation phase at the end before releasing the object. This was probably due to the fact that the amputated participants were more conscientious and wanted to have the object not bouncing out of the bucket, whereas the control participants did not bother about this.

Several reasons can be responsible for the longer durations and the higher number of actions needed for completing the task, the most important probably being the control delay induced by the classification of the PLM. Precise positioning of the segments of the prosthesis required a precise temporal control of the activation of the corresponding movements, which was clearly different between the tasks for the two groups. Indeed, the PLM-based control architecture induced additional delays of up to 512 ms because of the windowing and filtering effect of the classification algorithm. This clearly increased the difficulty to stop the elbow flexion at the desired height and possibly induced the flexion/extension oscillations observed for the elbow joint, shown in Figure 8 for the clothespin task. This delay can probably be decreased by the reduction of the sliding window time to 256 ms and the associated overlap to 64 ms, by the optimization of the set of features (the sample entropy being computationally time-consuming) and by using compiled programming languages.

Another reason for the reduced performance of the amputated participants is that the task was more complex for them (i.e., precise and reproducible mobilization of their phantom limb) than for the control participants (simply acting over a mouse), and, moreover, prolonged phantom limb mobilization induces fatigue (De Graaf et al., 2016), which influenced the sEMG signals and thus reduced the rate of successful classification of the PLM. This might have caused the increase of durations and number of actions for the ball and clothespin objects (which were presented after the cylinder). Finally, the patients reported that they had to concentrate when performing the PLM (specially to prevent from performing “unusual” corrective actions which would have perturbed the classifier) and to perform clear separations (i.e., a time of inactivity) between the different PLM to maximize the recognition rate. This also contributed to increasing the task durations. Yet, interestingly, even the durations of the control group are extended when compared to the time it takes to grasp and release the objects with an intact arm (below 5 s). This suggests that, more than the complexity of phantom limb mobilization, the impossibility of performing simultaneous actions is one of the main causes of the prolonged durations needed to perform these grasp-and-release tasks.

The obtained results show that amputated participants were able, after a very short appropriation of all task requirements (i.e., 2 repetitions for training, 8 preliminary repetitions of each PLM and two preliminary trials of each grasp-and-release task), to manage the rather complex interaction between their PLM, the associated actions of the prosthesis and, through it, their physical interaction with objects and the environment. The PLM-based control revealed to be rather intuitive. This is confirmed by the fact that the patients never used their PLM in daily life and still were able to control the prosthesis without any beforehand training. Moreover, the patients instinctively tried to correct unplanned actions generated at the prosthesis level with adapted phantom movements (as we do with intact limbs) but unfortunately not known by the classifier. This intrinsic limitation of the pattern recognition techniques that can only recognize specific and known (trained) PLM, still limits the intuitiveness and naturalness of the approach.

Obviously, in the current state of the control architecture, the control performance can be expected to be reduced with a worn prosthesis. Indeed, the current limited robustness of pattern recognition techniques is a major obstacle, particularly when they have to be used in realistic scenarios including a worn prosthesis. This will generate additional constraints such as pressure and sweating, along with a mobile residual limb generating non-“phantom limb related” muscle contractions. These factors will affect the sEMG signals and thus decrease the PLM detection rate. Yet, this is a generic problem in the field of pattern recognition of electro-physiological signals, and numerous solutions are actually developed that could compensate for the listed issues. Examples of these solutions are (1) robustness to electrodes shift (Muceli et al., 2014; He and Zhu, 2017), (2) use of osseointegration (Ortiz-Catalan et al., 2014) to eliminate the problem of the stump/socket physical connection, (3) electrode implantation (Mastinu et al., 2017) minimizing the issue with skin impedance and movements, and of course (4) more robust architectures of pattern recognition, integrating the stump posture (tracked through IMUs for example) to integrate the actual arm posture in the signal classification (Lauretti et al., 2016).

This work demonstrates the effectiveness of a bio-inspired system successfully conjugating the advantages of an underactuated, anthropomorphic hand with a PCA-based control strategy, and opens up promising possibilities for the development of an intuitively controllable hand prosthesis.

Despite the above-mentioned difficulties, our two patients were able, without any beforehand PLM-training, to control 8 different movements of a prosthesis in a more efficient, simple, and dexterous way that conventional (dual-site) myoelectric control can offer. While TMR is now becoming a generic solution for transhumeral amputees to increase their control capabilities over (or simply allow the use of) prosthesis with more than 2 active DoF (i.e., when an active elbow is added to the prosthetic wrist and hand), the preliminary results obtained in this study and other research teams exploiting the natural myoelectric activity related to PLM, pushes toward a reconsideration of the possibility of extracting more control signals without undergoing invasive surgical procedures. Obviously, TMR is providing an interesting way of stabilizing and reinforcing the myoelectric patterns related to PLM. This eases the decoding of myoelectric activities and is possibly one major key to overcome the issue of the perturbations generated by the wearing of the prosthesis. Nonetheless, recent “realistic” use of TMR in transhumeral amputees, controlling a 3-active-DoF-prosthesis through pattern recognition (Hargrove et al., 2017), still required either the use of a 15-electrodes-array placed over the stump (as described in Tkach et al., 2014), or the use of the conventional signal control switch to control the wrist when a direct control with a set of 4 electrodes was used. Therefore, there seems to be a real interest in pushing further our understanding of the phenomenon of natural (i.e., without TMS) residual myoelectric activity related to PLM. This could possibly be a viable option in some transhumeral amputees desiring to control 3 active joints.

The transhumeral amputees who were recruited in the study had received no prior training with the PLM-based-control approach. Better results, in terms of precision and completion times, can be expected with regular PLM-training. Furthermore, improving the control of the prosthetic joints (especially the elbow involved in numerous and rather long displacements), with exponential velocity and smoothened acceleration profiles to restore human-like movement properties (isochrony and minimization of the jerk (Viviani and Flash, 1995) will have a positive effect on the controllability. Bio-inspired approaches minimizing the control dimensionality through mechanical underactuation and models of finger joint synergies (as proposed by Magenes et al., 2008 or Matrone et al., 2010) could strongly enhance amputees' abilities in object manipulation tasks. Finally, providing additional sensory feedback to the participant, especially concerning the interaction of the prosthesis with the environment could be a game changer in helping amputated persons in performing such tasks, whatever control technique is used. For instance, artificially provoking phantom limb referred sensations, as recently tested in (Osborn et al., 2018), could be a relevant technique, particularly when prosthesis control is PLM-based. More amputated participants will be included in future experiments in order to reinforce the first results presented here and investigate the influence of phantom limb mobility training on the control performance. In such way, the often occurring mobility of the phantom limb might become useful instead of something to hide or ignore.

## Author contributions

NJ, EdM, JdG, CN, AT designed the protocol. EdM, FR, and NJ built the experimental platform, and EdM collected the data with the healthy participants. AT, NM, and JP contacted the amputated participant and organized the experimental session. NJ, CN, JdG, EdM, and AT participated in the experimental session with the amputated participant. NJ, JdG, CN, and AT analyzed the data, and wrote the present report.

### Conflict of interest statement

The authors declare that the research was conducted in the absence of any commercial or financial relationships that could be construed as a potential conflict of interest.

## References

[B1] Al-Timemy A. H. Bugmann G. Escudero J. Outram N. (2013). Classification of finger movements for the dexterous hand prosthesis control with surface electromyography. IEEE J. Biomed. Health Inform. 17, 608–618. 10.1109/JBHI.2013.2249590 24592463

[B2] André J. Paysant J. (2006). Les amputés en chiffres: épidémiologie. Module de MPR et Appareillage, co. fe mer avril. Available online at: www.cofemer.fr/UserFiles/File/AP2Amp_Chiffres.pdf

[B3] Atkins D. J. Heard D. C. Donovan W. H. (1996). Epidemiologic overview of individuals with upper-limb loss and their reported research priorities. J. Prosthet. Orthot. 8, 2–11. 10.1097/00008526-199600810-00003

[B4] Atzori M. Gijsberts A. Castellini C. Caputo B. Mittaz Hager A.-G. Elsig S. (2016). Clinical parameter effect on the capability to control myoelectric robotic prosthetic hands. J. Rehabil. Res. Dev. 53, 345–358. 10.1682/JRRD.2014.09.0218 27272750

[B5] Belter J. T. Dollar A. M. (2011). Performance characteristics of anthropomorphic prosthetic hands, in Rehabilitation Robotics (ICORR), 2011 IEEE International Conference on (Zurich: IEEE), 1–7. 10.1109/ICORR.2011.5975476 22275674

[B6] Biddiss E. Chau T. (2007). Upper-limb prosthetics: critical factors in device abandonment. Am. J. Phys. Med. Rehabil. 86, 977–987. 10.1097/PHM.0b013e3181587f6c 18090439

[B7] Castellini C. Artemiadis P. Wininger M. Ajoudani A. Alimusaj M. Bicchi A.. (2014). Proceedings of the first workshop on peripheral machine interfaces: going beyond traditional surface electromyography. Front. Neurorobot. 8:22. 10.3389/fnbot.2014.00022 25177292 PMC4133701

[B8] De Graaf J. B. Jarrassé N. Nicol C. Touillet A. Coyle T. Maynard L.. (2016). Phantom hand and wrist movements in upper limb amputees are slow but naturally controlled movements. Neuroscience 312, 48–57. 10.1016/j.neuroscience.2015.11.007 26556065

[B9] Englehart K. Hudgins B. Parker P. A. Stevenson M. (1999). Classification of the myoelectric signal using time-frequency based representations. Med. Eng. Phys. 21, 431–438. 10.1016/S1350-4533(99)00066-1 10624739

[B10] Farina D. Jiang N. Rehbaum H. Holobar A. Graimann B. Dietl H.. (2014). The extraction of neural information from the surface emg for the control of upper-limb prostheses: emerging avenues and challenges. IEEE Trans. Neural Syst. Rehabil. Eng., 22, 797–809. 10.1109/TNSRE.2014.2305111 24760934

[B11] Farthing J. P. Chilibeck P. D. (2003). The effects of eccentric and concentric training at different velocities on muscle hypertrophy. Eur. J. Appl. Physiol. 89, 578–586. 10.1007/s00421-003-0842-2 12756571

[B12] Finley F. R. Wirta R. W. (1967). Myocoder studies of multiple myopotential response. Arch. Phys. Med. Rehabil. 48:598. 6060789

[B13] Gagné M. Hétu S. Reilly K. Mercier C. (2011). The map is not the territory: motor system reorganization in upper limb amputees. Hum. Brain Mapp. 32, 509–519. 10.1002/hbm.21038 21391244 PMC6870038

[B14] Garbarini F. Bisio A. Biggio M. Pia L. Bove M. (2018). Motor sequence learning and intermanual transfer with a phantom limb. Cortex 101, 181–191. 10.1016/j.cortex.2018.01.011 29482016

[B15] Gaudet G. Raison M. Achiche S. (2018). Classification of upper limb phantom movements in transhumeral amputees using electromyographic and kinematic features. Eng. Appl. Artif. Intell. 68, 153–164. 10.1016/j.engappai.2017.10.017

[B16] Hargrove L. J. Miller L. A. Turner K. Kuiken T. A. (2017). Myoelectric pattern recognition outperforms direct control for transhumeral amputees with targeted muscle reinnervation: a randomized clinical trial. Sci. Rep. 7:13840. 10.1038/s41598-017-14386-w 29062019 PMC5653840

[B17] He J. Zhu X. (2017). Combining improved gray-level co-occurrence matrix with high density grid for myoelectric control robustness to electrode shift. IEEE Trans. Neural Syst. Rehabil. Eng. 25, 1539–1548. 10.1109/TNSRE.2016.2644264 28026779

[B18] Herberts P. Almström C. Kadefors R. Lawrence P. D. (1973). Hand prosthesis control via myoelectric patterns. Acta Orthopaed. Scand. 44, 389–409. 10.3109/17453677308989075 4771275

[B19] Hermens H. J. Freriks B. Disselhorst-Klug C. Rau G. (2000). Development of recommendations for semg sensors and sensor placement procedures. J. Electromyogr. Kinesiol. 10, 361–374. 10.1016/S1050-6411(00)00027-4 11018445

[B20] Jarrasse N. Nicol C. Richer F. Touillet A. Martinet N. Paysant J. (2017a). Voluntary phantom hand and finger movements in transhumerai amputees could be used to naturally control polydigital prostheses, in Rehabilitation Robotics (ICORR), 2017 International Conference on (London, UK: IEEE), 1239–1245. 10.1109/ICORR.2017.8009419 28813991

[B21] Jarrasse N. Nicol C. Touillet A. Richer F. Martinet N. Paysant J.. (2017b). Classification of phantom finger, hand, wrist, and elbow voluntary gestures in transhumeral amputees with semg. IEEE Trans. Neural Syst. Rehabil. Eng. 25, 71–80. 10.1109/TNSRE.2016.2563222 27164596

[B22] Kuiken T. A. Miller L. A. Lipschutz R. D. Lock B. A. Stubblefield K. Marasco P. D.. (2007). Targeted reinnervation for enhanced prosthetic arm function in a woman with a proximal amputation: a case study. Lancet 369, 371–380. 10.1016/S0140-6736(07)60193-7 17276777

[B23] Kvas G. Velik R. (2008). A filter approach for myoelectric channel selection, in 2008 6th IEEE International Conference on Industrial Informatics (Daejeon: IEEE), 1437–1440.

[B24] Lauretti C. Davalli A. Sacchetti R. Guglielmelli E. Zollo L. (2016). Fusion of m-imu and emg signals for the control of trans-humeral prostheses, in Biomedical Robotics and Biomechatronics (BioRob), 2016 6th IEEE International Conference on (Singapore: IEEE), 1123–1128.

[B25] Lawrence P. Herberts P. Kadefors R. (1973). Experiences with a multifunctional hand prosthesis controlled by myoelectric patterns. Adv. Ext. Control Hum. Extremit. 47–65.

[B26] Light C. M. Chappell P. H. Kyberd P. J. (2002). Establishing a standardized clinical assessment tool of pathologic and prosthetic hand function: normative data, reliability, and validity. Arch. Phys. Med. Rehabil. 83, 776–783. 10.1053/apmr.2002.32737 12048655

[B27] Magenes G. Passaglia F. Secco E. L. (2008). A new approach of multi-dof prosthetic control, in Engineering in Medicine and Biology Society, 2008. EMBS 2008. 30th Annual International Conference of the IEEE (Vancoucer, BC: IEEE), 3443–3446. 10.1109/IEMBS.2008.4649946 19163449

[B28] Mastinu E. Doguet P. Botquin Y. Håkansson B. Ortiz-Catalan M. (2017). Embedded system for prosthetic control using implanted neuromuscular interfaces accessed via an osseointegrated implant. IEEE Trans. Biomed. Circuits Syst. 11, 867–877. 10.1109/TBCAS.2017.2694710 28541915

[B29] Matrone G. C. Cipriani C. Secco E. L. Magenes G. Carrozza M. C. (2010). Principal components analysis based control of a multi-dof underactuated prosthetic hand. J. Neuroeng. Rehabil. 7:16. 10.1186/1743-0003-7-16 20416036 PMC2876164

[B30] Muceli S. Jiang N. Farina D. (2014). Extracting signals robust to electrode number and shift for online simultaneous and proportional myoelectric control by factorization algorithms. IEEE Trans. Neural Syst. Rehabil. Eng. 22, 623–633. 10.1109/TNSRE.2013.2282898 24132017

[B31] National Amputee Statistical Database (2009). The Amputee Statistical Database for the United Kingdom 2006/07. Edinburgh: Information Services Division, NHS Scotland.

[B32] Ortiz-Catalan M. Gudhmundsdóttir R. A. Kristoffersen M. B. Zepeda-Echavarria A. Caine-Winterberger K. Kulbacka-Ortiz K.. (2016). Phantom motor execution facilitated by machine learning and augmented reality as treatment for phantom limb pain: a single group, clinical trial in patients with chronic intractable phantom limb pain. Lancet 388, 2885–2894. 10.1016/S0140-6736(16)31598-7 27916234

[B33] Ortiz-Catalan M. Håkansson B. Brånemark R. (2014). An osseointegrated human-machine gateway for long-term sensory feedback and motor control of artificial limbs. Sci. Transl. Med. 6:257re6. 10.1126/scitranslmed.3008933 25298322

[B34] Osborn L. E. Dragomir A. Betthauser J. L. Hunt C. L. Nguyen H. H. Kaliki R. R. (2018). Prosthesis with neuromorphic multilayered e-dermis perceives touch and pain. Sci. Robot. 3:eaat3818 10.1126/scirobotics.aat3818 PMC7051004 32123782

[B35] Oskoei M. A. Hu H. (2008). Support vector machine-based classification scheme for myoelectric control applied to upper limb. IEEE Trans. Biomed. Eng. 55, 1956–1965. 10.1109/TBME.2008.919734 18632358

[B36] Ostlie K. Franklin R. J. Skjeldal O. H. Skrondal A. Magnus P. (2011). Musculoskeletal pain and overuse syndromes in adult acquired major upper-limb amputees. Arch. Phys. Med. Rehabil. 92, 1967–1973. 10.1016/j.apmr.2011.06.026 22133243

[B37] Phinyomark A. Quaine F. Charbonnier S. Serviere C. Tarpin-Bernard F. Laurillau Y. (2013). EMG feature evaluation for improving myoelectric pattern recognition robustness. Exp. Syst. Appl. 40, 4832–4840. 10.1016/j.eswa.2013.02.023

[B38] Powell M. A. Kaliki R. R. Thakor N. V. (2014). User training for pattern recognition-based myoelectric prostheses: improving phantom limb movement consistency and distinguishability. IEEE Trans. Neural Syst. Rehabil. Eng. 22, 522–532. 10.1109/TNSRE.2013.2279737 24122566 PMC10497233

[B39] Qi H. X. Stewart Phillips W. Kaas J. H. (2004). Connections of neurons in the lumbar ventral horn of spinal cord are altered after long-standing limb loss in a macaque monkey. Somatosens. Mot. Res. 21, 229–239. 10.1080/08990220400012588 15763908

[B40] Raffin E. Giraux P. Reilly K. T. (2012a). The moving phantom: motor execution or motor imagery? Cortex 48, 746–757. 10.1016/j.cortex.2011.02.003 21397901

[B41] Raffin E. Mattout J. Reilly K. T. Giraux P. (2012b). Disentangling motor execution from motor imagery with the phantom limb. Brain 135(Pt 2):582–595. 10.1093/brain/awr337 22345089

[B42] Reilly K. T. Mercier C. Schieber M. H. Sirigu A. (2006). Persistent hand motor commands in the amputees' brain. Brain 129, 2211–2223. 10.1093/brain/awl154 16799174

[B43] Resnik L. Klinger S. L. Etter K. (2014). The deka arm: its features, functionality, and evolution during the veterans affairs study to optimize the deka arm. Prosthet. Orthot. Int. 38, 492–504. 10.1177/0309364613506913 24150930

[B44] Richman J. S. Moorman J. R. (2000). Physiological time-series analysis using approximate entropy and sample entropy. Am. J. Physiol. Heart Circul. Physiol. 278, H2039–H2049. 10.1152/ajpheart.2000.278.6.H2039 10843903

[B45] Tkach D. Huang H. Kuiken T. (2010). Research study of stability of time-domain features for electromyographic pattern recognition. J. Neuroeng. Rehabil. 7:21 10.1186/1743-0003-7-21 20492713 PMC2881049

[B46] Tkach D. C. Young A. J. Smith L. H. Rouse E. J. Hargrove L. J. (2014). Real-time and offline performance of pattern recognition myoelectric control using a generic electrode grid with targeted muscle reinnervation patients. IEEE Trans. Neural Syst. Rehabil. Eng. 22, 727–734. 10.1109/TNSRE.2014.2302799 24760931

[B47] Touillet A. Peultier-Celli L. Nicol C. Jarrassé N. Loiret I. Martinet N.. (2018). Characteristics of phantom upper limb mobility encourage phantom-mobility-based prosthesis control. Nat. Sci. Rep. 8:15459. 10.1038/s41598-018-33643-0 30337602 PMC6193985

[B48] Viviani P. Flash T. (1995). Minimum-jerk, two-thirds power law, and isochrony: converging approaches to movement planning. J. Exp. Psychol. Hum. Percept. Perform. 21:32. 10.1037/0096-1523.21.1.32 7707032

[B49] Wirta R. W. Taylor D. R. Finley F. R. (1978). Pattern-recognition arm prosthesis: a historical perspective—a final report. Bull. Prosthet. Res. 10, 8–35. 365281

[B50] Wright T. W. Hagen A. D. Wood M. B. (1995). Prosthetic usage in major upper extremity amputations. J. Hand Surg. 20, 619–622. 10.1016/S0363-5023(05)80278-3 7594289

[B51] Wu C. W. Kaas J. H. (2000). Spinal cord atrophy and reorganization of motoneuron connections following long-standing limb loss in primates. Neuron 28, 967–978. 10.1016/S0896-6273(00)00167-7 11163280

[B52] Zardoshti-Kermani M. Wheeler B. Badie K. Hashemi R. (1995). Emg feature evaluation for movement control of upper extremity prostheses. IEEE Trans. Rehabil. Eng. 3, 324–333. 10.1109/86.481972

